# High Responsivity MgZnO Ultraviolet Thin-Film Phototransistor Developed Using Radio Frequency Sputtering

**DOI:** 10.3390/ma10020126

**Published:** 2017-02-04

**Authors:** Jyun-Yi Li, Sheng-Po Chang, Ming-Hung Hsu, Shoou-Jinn Chang

**Affiliations:** Department of Electrical Engineering and Advanced Optoelectronic Technology Center, Institute of Microelectronics, National Cheng Kung University, Tainan 701, Taiwan; z823040@gmail.com (J.-Y.L.); hsuminghung0121@gmail.com (M.-H.H.); changsj@mail.ncku.edu.tw (S.-J.C.)

**Keywords:** magnesium zinc oxide, ultraviolet, thin-film transistor, phototransistor

## Abstract

We investigated the electrical and optoelectronic properties of a magnesium zinc oxide thin-film phototransistor. We fabricate an ultraviolet phototransistor by using a wide-bandgap MgZnO thin film as the active layer material of the thin film transistor (TFT). The fabricated device demonstrated a threshold voltage of 3.1 V, on–off current ratio of 10^5^, subthreshold swing of 0.8 V/decade, and mobility of 5 cm^2^/V·s in a dark environment. As a UV photodetector, the responsivity of the device was 3.12 A/W, and the rejection ratio was 6.55 × 10^5^ at a gate bias of −5 V under 290 nm illumination.

## 1. Introduction

In recent years, ultraviolet photodetectors have attracted much attention for their potential in medical, commercial, and military applications [1,2,3,4,5]. The thin-film transistor (TFT) has been intensively researched for its application to switching devices in large-area display panels (active matrix liquid crystal displays (AMLCDs) and organic light-emitting diodes). A number of different materials are utilized for TFT fabrication. Many groups have used ZnO-based semiconductors as the TFT channel layer, owing to their high field mobility, low temperature processing, and nontoxicity. Oxide TFTs with photosensitive metal oxide semiconductor materials are promising candidates that can act as photodetectors. Bae et al. reported the fabrication of a ZnO-based phototransistor [6]. Zan et al. reported on an amorphous IGZO (a-IGZO) visible-light photodetector with a polymeric light absorption layer [7]. Chiu et al. reported the fabrication of deep-UV-sensitive a-IGZO TFTs with a Ta_2_O_5_ gate dielectric [8]. However, the electrical properties and material characteristics of the TFT’s active layer strongly influence the device’s transfer characteristics. TFTs with a ZnO active layer exhibit n-type conductivity owing to the oxygen vacancies and zinc interstitials. Thus, defect density control is the key to the performance of ZnO-based TFTs [9,10,11,12]. Magnesium zinc oxide is emerging as a TFT active layer candidate because its large bandgap can decrease donor-like defects [13,14]. In addition, the strong bonding energy between magnesium and oxide reduces oxygen vacancies, which makes Mg a good candidate for doping into ZnO for the TFT active layer. In several studies, TFTs with MgZnO have been fabricated with various processing methods, such as radio frequency-sputtering with a 5% Mg target [15], e-beam evaporation with 1% Mg content [16], and atomic layer deposition with 10% Mg content [17]. Because of its wide direct bandgap, the MgZnO material system is an excellent choice for optoelectronic devices in the UV portion of the spectrum. MgZnO also possesses unique figures of merit such as intrinsic visible blindness and radiation hardness that are crucial for practical optoelectronic devices. Hullavarad et al. reported that the UV/visible rejection ratio—defined as the ratio of the photoresponse at 310–800 nm—is 10^4^ for Mg_0.15_Zn_0.85_O on Al_2_O_3_ and 10^3^ on quartz substrates. In addition, solar or visible–blind Mg_x_Zn_1−x_O photodetectors have been fabricated on sapphire, glass, and silicon substrates [18]. Photodetectors fabricated on Mg_0.68_Zn_0.32_O/SrTiO_3_/Si have demonstrated the peak photoresponse at 225 nm and a UV/visible rejection ratio that is only one order of magnitude [19]. Prototype Mg_x_Zn_1−x_O UV photodetectors with different Mg contents have been fabricated with high photoresponsivities and sharp cutoffs at ~375, ~350, ~315, and ~300 nm for *x* values of 0, 0.10, 0.26, and 0.34, respectively [20]. Magnesium doping suppresses the subthreshold current. Furthermore, the magnesium atom also exists at interstitials in the crystal and forms impurity scattering centers, which leads to poor TFT mobility and a large threshold voltage. Nonetheless, there have been few studies related to MgZnO TFTs, let alone their fabrication via radio frequency-sputtering.

In this work, we investigated the properties of MgZnO TFTs that are fabricated by radio frequency-sputtering with different oxygen flow ratios, and then generalized the optimized conditions. We extended the application of the TFTs to a phototransistor to combine the photoelectrical properties of MgZnO and the transistor to increase the responsivity. Under the optimal parameters, the MgZnO TFT can operate normally, and the device has high mobility, a fast on–off transition, and high responsivity under deep UV illumination.

## 2. Materials and Methods

**Figure 1:**
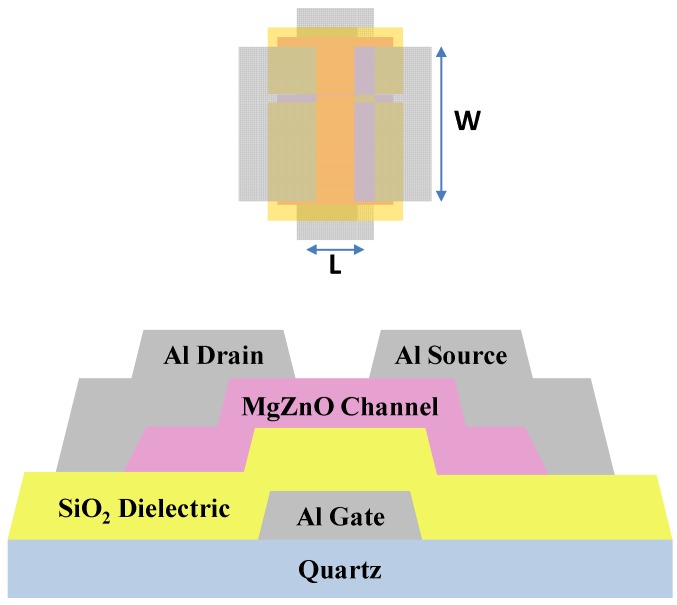
Top view and cross-section of the MgZnO thin-film transistor (TFT). W, channel width; L, channel length.

First, to fabricate MgZnO TFTs, 2 cm × 2 cm glass substrates were cleaned with acetone, isopropyl alcohol, and deionized (DI) water. Figure 1 shows the cross-section of the MgZnO TFT. The aluminum bottom gate was thermally deposited on a quartz substrate (Sunmei Glass Company, Taiwan, China). Next, a 200 nm thick SiO_2_ layer—which acted as a dielectric—was deposited by using the plasma-enhanced chemical vapor deposition (PECVD) process (PD-220NA, SAMCO, Kyoto, Japan). The 10-nm-thick MgZnO channel was fabricated from a MgZnO (MgO = 10 wt%, ZnO = 90 wt%) target (GfE Gesellschaft für Elektrometallurgie mbH: GfE, Nürnberg, Germany). During channel layer sputtering, the chamber pressure was kept at 10 mTorr, the sputtering power was fixed at 100 W, and the substrates were rotated at a speed of 20 rpm. The oxygen flow ratio was varied from 0% to 21% in increments of 7% (i.e., 0%, 7%, 14%, and 21%). After the channel layer deposition, the samples were placed in a furnace for annealing for 30 min at 300 °C. This was done to incorporate the magnesium atoms into the crystal lattice. The source and drain electrodes were deposited on the MgZnO active layer by thermal evaporation. The width/length (W/L) of the active layer was fixed at 1000 µm/100 µm. The current-voltage (I–V) characteristics of the fabricated TFTs were measured in the dark at room temperature and atmospheric pressure with a B1500 semiconductor parameter analyzer (Agilent Technologies, Santa Clara, CA, USA). The parameters of the TFTs were calculated by using Equation (1).

The mobility of our TFTs was determined in the saturation region. In the saturation region, the drain current can be represented by

(1) $$I_{D}=\frac{W}{2L}C\mu {\left(V_{G}-V_{t}\right)}^{2},$$

where *C* is the capacitance of the dielectric layer (~16.8 nF·cm^−2^); *W* is the channel width; *L* is the channel length; *V_t_* and *V_G_* are the threshold voltage and gate voltage respectively; and *µ* is the field-effect mobility. We used the gate width-length (W/L) ratio of 10. The subthreshold swing (*S.S.*) is defined as

(2) $$S.S.=\frac{\partial V_{G}}{\partial \log I_{D}}\;,$$

where *V_G_* and *I_D_* are the gate voltage and drain current respectively. The small value of *S.S.* was attributed to both the high gate capacitance density and high interface charge density. The photoresponsivity of the fabricated device was measured with a 250 W Xe lamp dispersed by a monochromator as the light source. The monochromatic light, which was calibrated with a UV-enhanced Si diode and optical power meter, was modulated by a mechanical chopper and then collimated on the front side of the fabricated device with an optical fiber. The illumination area was 0.1 mm^2^.

## 3. Results and Discussion

**Figure 2:**
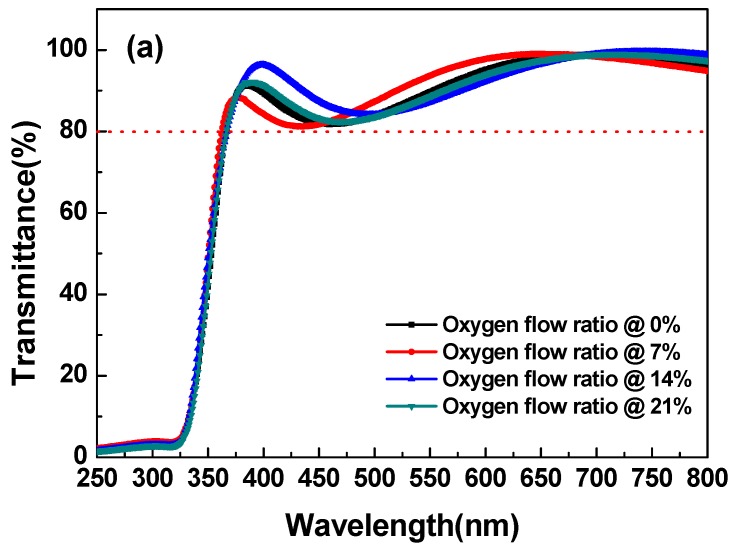

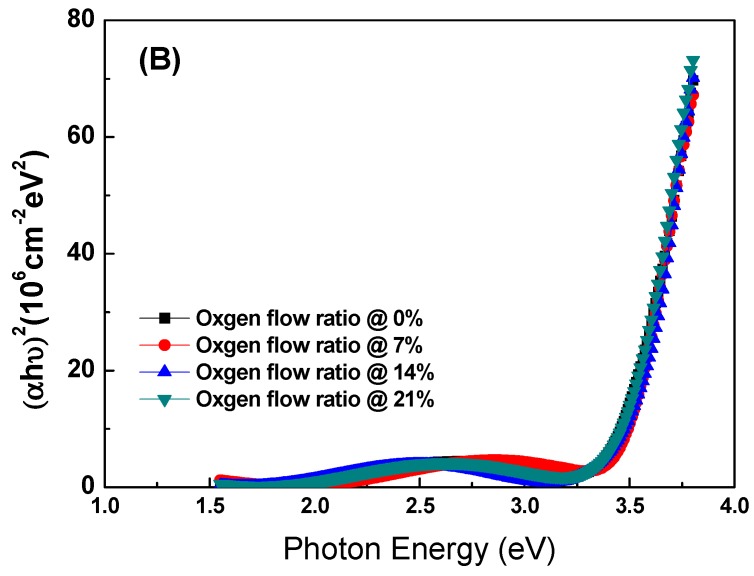
(**A**) Transmittances of the MgZnO thin film with a variable oxygen flow ratio of 0%–21%, (**B**) Absorption coefficient versus the photon energy for MgZnO thin film with a variable oxygen flow ratio of 0%–21%.

As for the film optical properties, the transmittance and optical bandgap determined by the absorption coefficient were considered. Figure 2 shows the transmittance spectra of the MgZnO thin film with various oxygen flow ratios. The transmittance in the visible region could clearly be more than 80%. The absorption edge of the MgZnO thin film was from 329 nm to 334 nm. The inset depicts the relationship between absorption coefficient and photon energy. The energy bandgap of MgZnO was found to be around 3.43 eV, regardless of the oxygen flow ratio.

The variation in the oxygen ratio hardly affected the absorption edge. When oxygen gas was not introduced during sputtering, more defects developed in the MgZnO thin film. Accordingly, changes in the sputtering oxygen flow ratio were considered to determine the compensation level of the oxygen vacancies.

**Figure 3:**
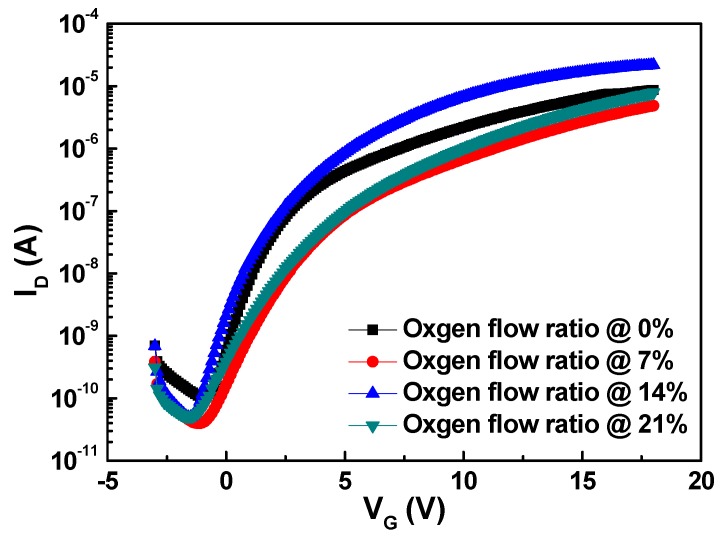
Transfer characteristics of the MgZnO TFT.

Figure 3 shows the transfer characteristics of the TFTs with various oxygen flow ratios, and Table 1 lists the parameters. Table 1 indicates that samples with an oxygen flow ratio of 14% exhibit the best characteristics. The MgZnO TFT with a 0% oxygen flow ratio had more defects compared with the other samples, leading to poor electrical properties. When the oxygen flow ratio increased, the properties were gradually improved. The oxygen flow ratio of 14% reduced the oxygen vacancies in the crystal properly however, the oxygen flow ratio of 21% caused the formation of acceptor-like defects owing to the excessive oxygen. This conclusion can also be obtained from the difference in *S.S.* for various oxygen flow ratios. When the flow ratio was 14%, the TFT had the lowest *S.S.* and a relatively small total trap density. At an ideal oxygen flow ratio, the on–off ratio could be up to five orders of magnitude.

**Figure 4:**
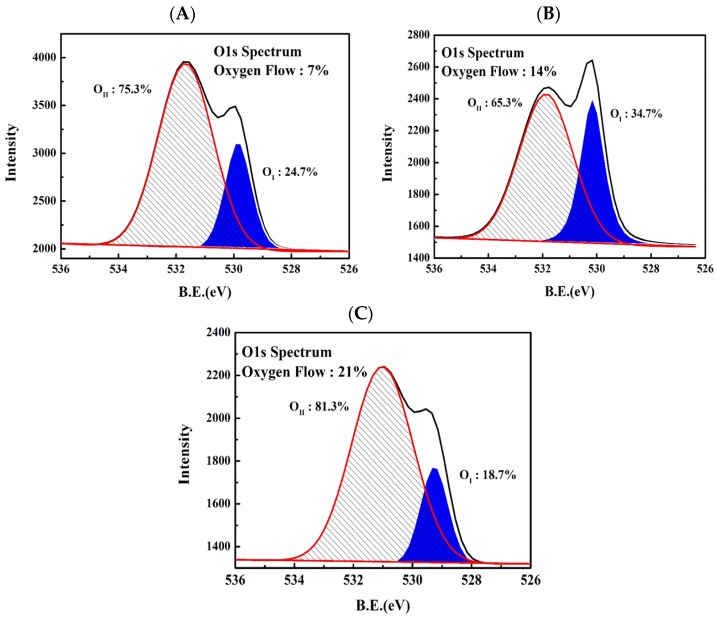
X-ray photoelectron spectroscopy (XPS) O 1s spectrum of a MgZnO thin film sputtered with oxygen flow ratios of (**A**) 7%; (**B**) 14%; and (**C**) 21%.

Figure 4 shows the XPS spectra of O 1s for films grown at different oxygen flow ratios from 7% to 21%. The peak at the lower binding energy of ~530 eV (O_I_) was attributed to O^2−^ ions present in a stoichiometric wurtzite MgZnO structure. The peak at the higher binding energy of ~532 eV (O_II_) was is attributed to oxygen deficiencies in MgZnO [21]. This result shows that, as the flow ratio increased from 7% to 14% the oxygen in the films tended to compensate for the vacancies in the crystal lattice. This can be confirmed by the spectrum curve area decreasing from 75.3% to 65.3%. However, as the sputtering oxygen flow ratio was increased to 21%, the oxygen composition in the film became defective, which contributed to undesirable oxygen interstitials. The spectrum area at the higher binding energy increased from 65.3% to 81.3%, which is related to the difference in the *S.S.* of various oxygen flow ratios. When the flow ratio was 14%, the TFT had the lowest *S.S.* and a relatively low total trap density.

**Figure 5:**
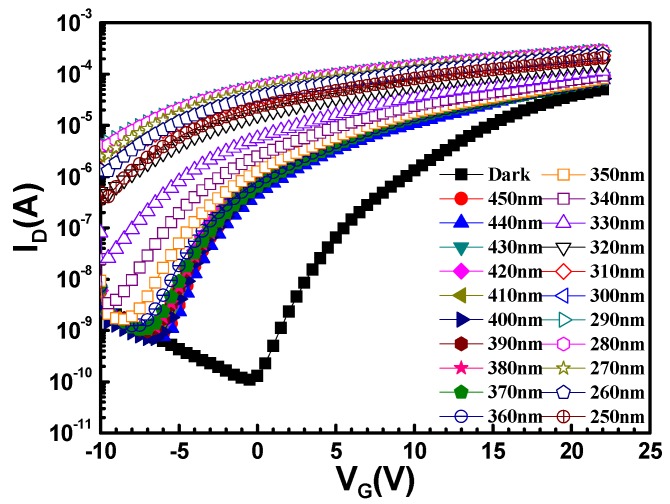
*I_D_*–*V_G_* output characteristics of an MgZnO thin-film phototransistor under illumination.

Oxide TFTs with photosensitive metal oxide semiconductor materials can be used as phototransistors, which are key components in optoelectronic circuits. Hence, we exposed the MgZnO TFTs to light to analyze the photo characteristics. Figure 5 shows the transfer characteristics for the oxygen flow ratio of 14% for an MgZnO TFT in the dark and under illumination from 450 nm to 250 nm when plotted as a function of *V_G_* from −10 V to 25 V, with drain voltage (*V_D_*) fixed at 12 V. Under illumination, the threshold voltage had a significant negative shift, and the drain current increased owing to the photo-generation from excessive holes and free electrons. Therefore, *I_d_* of the MgZnO TFT illuminated with UV light (whose photon energy is greater than the bandgap of the semiconducting material) has two components: (i) the current flowing between the source/drain electrodes (*I_ds_*) because of the applied bias voltage, and (ii) the photoconductive component (*I_ph_*):

(3) $$I_{d}=I_{ds}+I_{ph}.$$

*I_ph_* is given by the relation

(4) $$I_{ph}=\frac{q\left(\mu_{n}\right)\eta F_{ph}\tau_{p}WV_{DS}}{L},$$

where *µ_n_* is electron mobility, *η* is the quantum efficiency, *F_ph_* is the photon flux, *τ_p_* is the carrier lifetime, *q* is the electronic charge, *W* and *L* are the width and length of the device, respectively, and *V_DS_* is the voltage applied between the source and drain electrodes [22]. Thus, for a given photon flux *F_ph_*, the photoconductive component will be large if *V_DS_* is large. Hence, with the corresponding threshold voltage shift, the transistor is easier to turn on. The drain current was enhanced in the wavelength range between 300 and 350 nm, which corresponds to the optical characteristics of MgZnO.

**Figure 6:**
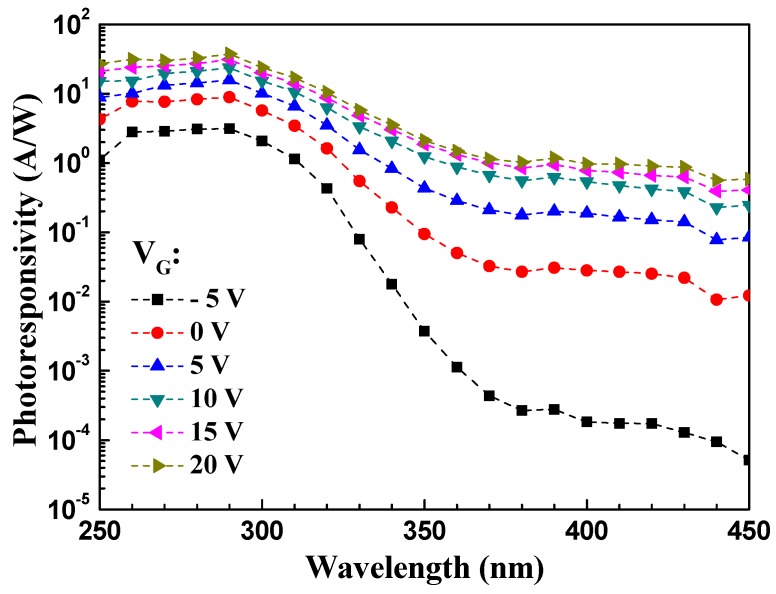
Photoresponsivity spectra of the MgZnO thin-film phototransistor with a bias of −5 V to 20 V under different light illumination.

Figure 6 shows the spectral response of the fabricated MgZnO device. Here, we define the UV-to-visible rejection ratio as the responsivity measured at 290 nm divided by the responsivity measured at 450 nm:

(5) $$Rejection\;Ratio\;\left(R.R.\right)=\frac{Responsivity\;\left(290\;\mathrm{nm}\right)}{Responsivity\;\left(450\;\mathrm{nm}\right)}.$$

UV-to-visible rejection ratio of a photodetector measures the ability of a device to detect UV light signals compared to visible light signals. The value of UV-to-visible rejection ratio is the responsivity of a certain wavelength of UV light region divided by the responsivity of a certain wavelength of visible light region. If the rejection ratio is high, it implies that the device is more sensitive to UV light. We pursue the goal of accomplishing a photodetector with high UV-to-visible rejection ratio. With an incident light wavelength of 290 nm and an applied gate bias of −5 V, the measured responsivity of the device was 3.12 A/W, and the UV-to-visible rejection ratio was 6.55 × 10^5^. Such result again indicates that the fabricated TFT was very UV-sensitive and can be used as a solar-blind phototransistor.

## 4. Conclusions

The best characteristics of the MgZnO TFT with a SiO_2_ dielectric were obtained with a sputtering oxygen flow rate controlled at 14% in order to compensate for the oxygen vacancies. Under this condition, the transistor exhibited an on–off ratio of five orders of magnitude, and a mobility of ~5 cm^2^/Vs was achieved. Our results broaden the applicability of MgZnO TFTs to photodetectors. When a negative gate bias was applied, the optimized MgZnO phototransistor exhibited fine photo properties. The responsivity of the device was 3.12 A/W, and the rejection ratio was 6.55 × 10^5^ at a gate bias of −5 V under 290 nm illuminations. The results indicate that the fabricated device is suitable for solar-blind photodetectors.

## Figures and Tables

**Table 1:** Transfer characteristics of the MgZnO TFT with various oxygen ratios.

Oxygen Ratio	*V_t_* (V)	*µ*_eff_ (cm^2^/Vs)	On–Off Current Ratio	*S.S.*	*N_t_*
0%	3.6 ± 0.072	2.42 ± 0.048	8.4 × 10^4^ ± 1680	0.89 ± 0.018	1.6 × 10^12^
7%	6.6 ± 0.132	2.17 ± 0.043	1.2 × 10^5^ ± 2400	1.65 ± 0.033	2.9 × 10^12^
14%	3.1 ± 0.062	5.65 ± 0.113	4.4 × 10^5^ ± 8800	0.80 ± 0.016	1.4 × 10^12^
21%	6.2 ± 0.124	3.25 ± 0.065	1.5 × 10^5^ ± 3300	1.36 ± 0.027	2.4 × 10^12^

*V_t_*, threshold voltage; *µ*_eff_, field-effect mobility; *S.S.*, subthreshold swing; *N_t_*, trapping density.
